# Analysis of the Impact of Comorbidities on Endometrial Lesions Using the Charlson Comorbidity Index in Western Romania

**DOI:** 10.3390/medicina57090945

**Published:** 2021-09-08

**Authors:** Alexandru Furau, Delia Mirela Tit, Cristian Furau, Simona Bungau, Gheorghe Furau, Mirela Marioara Toma, Catalin Gabriel Cirstoveanu, Izabella Petre, Denisia-Suzana Todor, Radu Stefan Romosan, Marius Craina

**Affiliations:** 1 Department of Oncology, “Vasile Goldis” Western University of Arad, 310414 Arad, Romania; marius.furau@yahoo.com; 2 Doctoral School of Medicine, “Victor Babes” University of Medicine and Pharmacy, 300041 Timisoara, Romania; 3 Department of Pharmacy, Faculty of Medicine and Pharmacy, University of Oradea, 410028 Oradea, Romania; mirela_tit@yahoo.com (D.M.T.); mire.toma@yahoo.com (M.M.T.); 4 Doctoral School of Biological and Biomedical Sciences, University of Oradea, 410087 Oradea, Romania; 5 Department of Pathophysiology, Faculty of Medicine, “Vasile Goldis” Western University of Arad, 310414 Arad, Romania; cristianfurau@gmail.com (C.F.); denimafti@gmail.com (D.-S.T.); 6 Department of Obstetrics and Gynaecology, Emergency Clinical County Hospital of Arad, 310037 Arad, Romania; gfurau@yahoo.com; 7 Department of Obstetrics and Gynaecology, “Vasile Goldis” Western University of Arad, 310414 Arad, Romania; 8 Department of Paediatrics, “Carol Davila” University of Medicine and Pharmacy, 020021 Bucharest, Romania; catalin.cirstoveanu@umfcd.ro; 9 Department of Obstetrics and Gynaecology, Faculty of Medicine and Pharmacy, “Victor Babes” University of Medicine and Pharmacy, 300041 Timisoara, Romania; dr.petreizabella@yahoo.com (I.P.); mariuscraina@hotmail.com (M.C.); 10 Department of Neurosciences-Psychiatry, “Victor Babes” University of Medicine and Pharmacy, 300041 Timisoara, Romania; romosan.radu@gmail.com; 11 Centre for Cognitive Research in Neuropsychiatric Pathology, “Victor Babes” University of Medicine and Pharmacy, 300041 Timisoara, Romania

**Keywords:** endometrial lesions, Charlson comorbidity index, endometrial cancer, endometrial hyperplasia

## Abstract

*Background and Objectives:* This retrospective study aimed to identify the main comorbidities found in gynecological patients hospitalized for endometrial lesions and to analyze the relationships between these comorbidities and each type of endometrial lesion. The Charlson comorbidity index (CCI) was calculated, thus assessing the patient’s probability of survival in relation to the underlying disease and the existing comorbidities. *Materials and Methods*: During 2015–2019, 594 cases hospitalized for vaginal bleeding outside of pregnancy were included in the research. For all cases, the frequency of comorbidities was calculated, applying the Cox proportional hazard model, considering the hospitalizations (from the following year after the first outpatient or hospital assessment) as a dependent variable; age and comorbidities were considered as independent variables. *Results*: Analysis of variance (ANOVA) for mean age of patients enrolled after diagnosis and multiple comparisons (via the Tukey post-hoc test) indicate significant differences (*p* < 0.05) between the average age for endometrial cancer (EC) and that for the typical endometrial hyperplasia or other diagnoses. The most common comorbidities were hypertension (62.28%), obesity (35.01%), and diabetes (22.89%), followed by cardiovascular disease. An intensely negative correlation (r = −0.715281634) was obtained between the percentage values of comorbidities present in EC and other endometrial lesions. The lowest chances of survival were calculated for 88 (14.81% of the total) patients over 50 years (the probability of survival in the next 10 years being between 0 and 21%). The chances of survival at 10 years are moderately negatively correlated with age (sample size = 594, r = −0.6706, *p* < 0.0001, 95% confidence interval (CI) for r having values from −0.7126 to −0.6238) and strongly negatively correlated with the CCI (r = −0.9359, *p* < 0.0001, 95% CI for r being in the range −0.9452 to −0.9251). *Conclusions*: Using CCI in endometrial lesions is necessary to compare the estimated risk of EC mortality with other medical conditions.

## 1. Introduction

The latest data from 2018 (published in May 2019) on neoplastic disease in Romania, according to Globocan 2018 [1], show that pathology due to endometrial cancer (EC) ranks 11th in the hierarchy, with 2470 new cases, representing 3% of all cancers in 2018, with 510 deaths (meaning 1% of all cancer deaths in Romania in 2018, 21st place). Although there is remarkable progress in the survival of patients with a particular type of neoplastic disease, mortality continues to rise in other situations, such as uterine cancer [2].

Worldwide life expectancy in women has been increasing [3], as well as comorbidities related to age [4]. Elderly women generally present EC, and adequate cancer therapy cannot be applied in many cases due to patients’ multiple medical comorbidities [5,6]. Tolerance to treatment, as well as survival outcome in EC, is affected by advanced age, cardiovascular disorders, diabetes mellitus (DM), and other components of metabolic syndrome [7,8,9,10].

Although the link between EC and metabolic syndrome is proven, the mechanisms by which metabolic syndrome causes EC are undetermined; this may be due to elevated serum values of metabolites (glucose, insulin, insulin-like growth factor, and triglycerides). Epidemiological studies have shown that type 2 DM doubles the risk for EC, being strongly correlated with age, but also with increased specific mortality and non-cancer-related mortality in women with EC [11]. The influence of comorbidities in women with gynecologic cancer and the negative impact on survival endpoints that they have was highlighted by countless researchers in ovarian carcinoma and early-stage EC [12,13,14,15].

This retrospective study aimed at the following: identification of the main comorbidities observed in gynecological patients with hospitalization for endometrial lesions; analysis of the relationships that these comorbidities establish with each type of endometrial lesion, for each being calculated the Charlson comorbidity index (CCI); probabilistic assessment of patient survival related to the underlying disease and comorbidities present; identifying the chances of 10-year survival of these patients; assessing the impact that comorbidities have on the evolution of a gynecological patient; highlighting the most common associated diseases involved.

## 2. Materials and Methods

### 2.1. Study Design

This bicentric observational retrospective study includes 594 cases, admitted in the obstetrics and gynecology department for vaginal bleeding, in two county emergency hospitals (namely Arad and Timisoara), located in western Romania, between 2015 and 2019. Condition for inclusion in the study: continuous hospitalization in an obstetrics-gynecology unit for uterine bleeding, outside pregnancy. Exclusion criteria were vaginal bleedings as an incident during pregnancy, postabortion, or postpartum period.

For all cases, the frequency of comorbidities was calculated, applying the Cox proportional hazard model for hospitalizations occurring in the following year from the first outpatient or hospital assessment, as a dependent variable, with independent variables being age and comorbidities. The evaluation was staged, proceeding with the use of the first comorbidity, as an independent variable, followed by the second and the others, until quantifying all recorded comorbidities.

### 2.2. Charlson Comorbidity Index in Endometrial Lesions

The CCI (as a method of categorizing comorbidities) analyzes patients’ secondary diagnoses, which are coded in the administrative data of healthcare providers according to the International Classification of Diseases; each of the comorbidities is associated with a value from 1 to 6, depending on the adjusted risk of death for it, and the sum of these values represents the score of the patient with comorbidities. The zero value of the score means the absence of a comorbidity, and the higher the score, the more probable the mortality.

The CCI covers both the aspect of the number of comorbidities present in addition to the underlying disease, as well as the severity of these comorbidities, representing a score that can predict short- or long-term death, depending on the length of hospitalization, impaired physiological function, and mortality rates.

In compiling CCI:

- Comorbidities are organized into 16 categories, but six of them cannot be established from the medical documents of the patients (myocardial infarction, COPD, collagenases, leukemia, lymphoma, and AIDS);
- It does not include obesity, hypertension (HT) (but only complications from myocardial infarction, chronic kidney disease, etc.), venous circulation disorders, asthma, and bronchopneumonia, nor locomotor, sensory, and cognitive deficiencies, which is why they have been analyzed separately.

The patients in the study were grouped into four categories according to the assigned scores, the zero score being of control [16].

### 2.3. Statistical Analysis

The hazard ratios (HR) were used (according to the Cox model of proportional hazard) with the revision of the CCI according to the principle: value 1 for comorbidities with HR adjusted between 1.2 and 1.5; value 2 for HR between 1.5 and 2.5; value 3 for HR between 2.5 and 3.5; value 4 for HR between 3.5 and 4.5; value 5 for HR above 6 (because comorbidities cannot have values greater than or equal to 4.5 but less than 6). Each variable has a dedicated score. For validation was used the MedCalc program, which gives the clinician the possibility to quickly calculate the estimated survival at 10 years based on the value of the CCI [16], which considers:

- Age, based on 5 age categories (0 = under 50, 1 = 50–59, 2 = 60–69, 3 = 70–79, 4 = over 80), where an additional point is assigned for each decade of life for the age over 50, maximum 4 points;
- The existence of myocardial infarction (MI) in the patient’s history, defined or probable, established as a result of an electrocardiogram (EKG) and/or enzymatic changes;
- Congestive heart failure: the existence of nocturnal paroxysmal dyspnea episodes that responded to digitalis, diuretics, etc.;
- Peripheral arterial disease (PAD) manifested by intermittent claudication, history of gangrene or arterial insufficiency, or the presence of an untreated abdominal or thoracic aneurysm (≥6 cm);
- Personal pathological history of stroke/including transient ischemic attack, chronic cognitive deficits, chronic obstructive pulmonary disease (COPD), collagenases, peptic ulcer disease, liver disease (severe—cirrhosis and portal HT with a history of rupture of esophageal varices, moderate—cirrhosis and portal HT without a history of rupture of esophageal varices, mild—chronic hepatitis or cirrhosis without portal HT), DM (without or controlled by diet, complicated or uncomplicated), hemiplegia, chronic kidney disease (moderate—creatinine below 3 mg/dL (0.27 mmol/L) or severe—on dialysis, posttransplant status, uremia), solid tumor (localized or metastatic), leukemia, lymphoma, acquired immunodeficiency syndrome (AIDS).

By attributing one point to each decade of age over 50 in which the patient falls, and calculating individual CCI, the chances of survival in the next 10 years were obtained, statistical validation resulting after both performing the Chi-square test and analyzing the ANOVA variant. MedCalc^®^ 14, IBM^®^ SPSS^®^ Statistics version 24 and Microsoft Excel 365^®^ were used as statistical software.

## 3. Results

Of the 594 cases analyzed, 153 were EC (25.75%), and 147 were endometrial hyperplasia (EH) (24.74%), the majority, 133, with hyperplasia without atypia (90.47%) and 14 having atypical hyperplasia/endometrioid intraepithelial neoplasia.

Analysis of variance (ANOVA) for mean ages of patients included after diagnosis (ECs, hyperplasia without atypia, atypical hyperplasia, and other diagnoses), and multiple comparisons (via Tukey post-hoc test) indicate significant differences, as Table 1 shows:

- Between the average age in EC and that in the typical EH, with the main specification that at higher average age, there are malignant lesions, followed by benign ones, at a distance of at least a decade;
- Between the average age in EC and other diagnoses, with the main specification that malignant lesions are present at higher average age, while other diagnoses of benign endometrial diseases are of particular interest to women with average age of at least one decade less;
- Between the average age in atypical EH and that in typical EH with the main specification that at higher average age, there are premalignant lesions due to atypical EH, while other typical EH are of particular interest in women with an average age smaller by at least a decade;
- Between the mean age in typical EH and that in other diagnoses.

The most common comorbidities recorded for the patients in the study were HT (62.28%), obesity (35.01%), and diabetes (22.89%), followed by cardiovascular disease, as Table 2 presents.

Single or associated comorbidities have been identified as part of the CCI, the most common being: 40 cases with localized solid tumors (6.73%) in the breast, lung, etc., and 2 cases with metastases (0.34%); 50 cases presented mild hepatic impairment (8.42%) and 7 cases with moderate to severe hepatic impairment (1.18%); 40 cases had stroke or transient ischemic attacks (6.73%); 104 cases were with uncomplicated diabetes (17.51%); 29 cases of DM with organ failure (4.88%); 31 cases with congestive heart failure (5.22%). The exclusive analysis of comorbidities of the enrolled cases, on EC, atypical EH, and other typical atypical EH; all these above mentioned are illustrated in Table 3, considering Charlson criteria.

**Figure 1:**
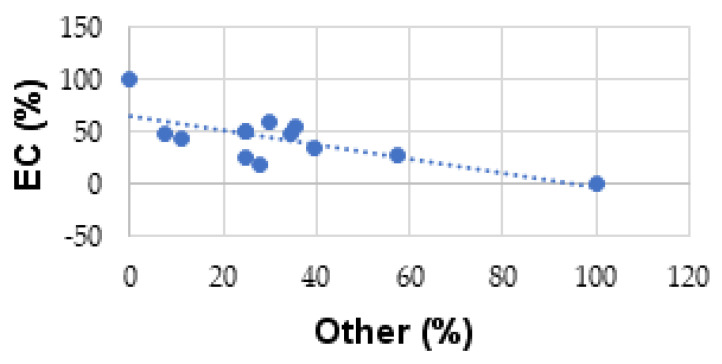
Correlation of comorbidities in endometrial cancer (EC) and other lesions.

From Table 3, an intensely negative correlation has been obtained (r = −0.715281634) between the percentage values of comorbidities present in EC and other endometrial lesions (Figure 1).

The lowest chances of survival were calculated for 88 (14.81% of the total) patients over 50 years (the probability of survival in the next 10 years being between 0 and 21%). A number of 161 (21.10%) patients have chances between 53 and 77% to survive 10 years, and 215 subjects (36.37%) have >90% chances of 10 years’ survival.

**Figure 2:**
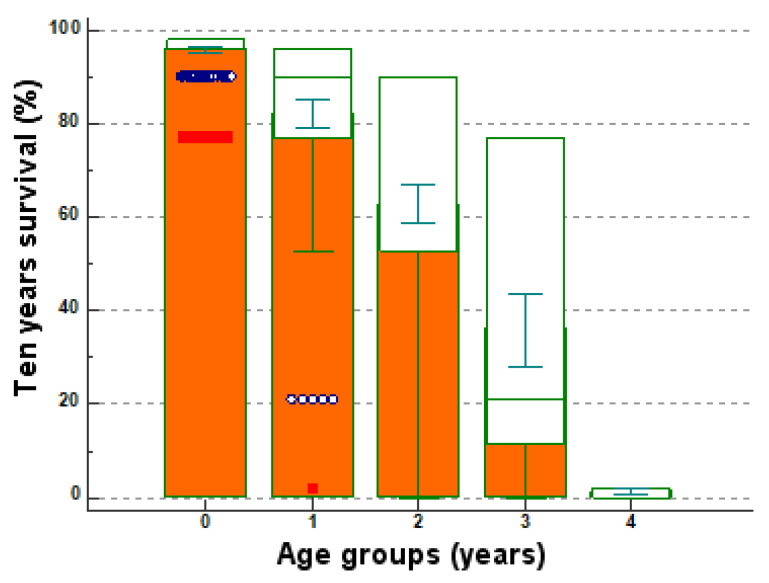
The chance of survival at 10 years by age categories (multiple comparison graph). Legend: 0 for <50, 1 for 50–59, 2 for 60–69, 3 for 70–79, 4 for >80; green intervals—error bars and represents 95% confidence intervals; blue circles—cases that deviate from the average; red bars—case averages by age categories in the legend.

In general, the patients < 50 years of age (*n* = 196) have an average of 95.8571% chances to survive 10 years (SD 4.3712); these chances decrease to 82.3125% (SD 19.0037) for the decade of age 50–59 years (*n* = 144); decrease to 62.9061% (SD 28.3764) for the decade of age 60–69 years (*n* = 181); decrease to 35.9833% for the decade 70–79 years (SD 30.5811); decrease to 1.3846% (SD 0.9608) for patients > 80 years (*n* = 13) (Figure 2).

**Figure 3:**
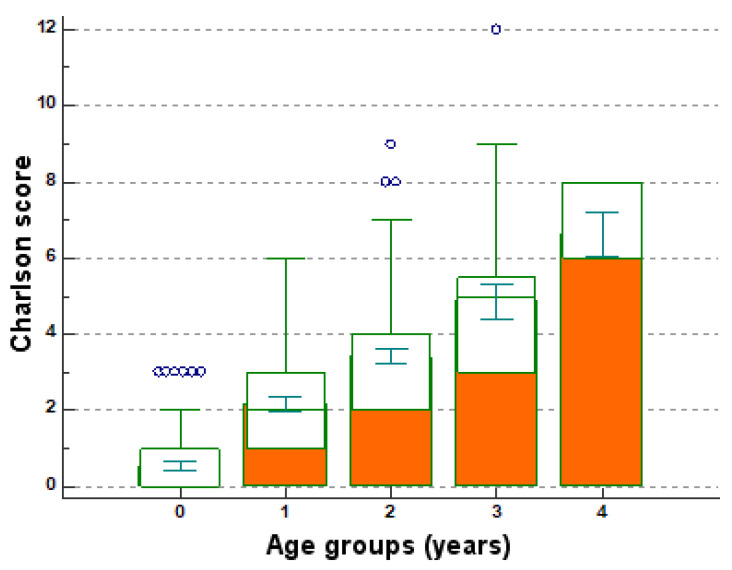
Charlson scores and age categories (Multiple comparison graph). Legend: 0 for <50, 1 for 50–59, 2 for 60–69, 3 for 70–79, 4 for >80; green intervals—error bars and represents 95% confidence intervals; blue circles—cases that deviate from the average.

The variance of Charlson scores (between 0 and 12) in relation to the chances of ten-year survival (between 0 and 98%) (Table 4, Table 5 and Figure 3) obviously shows the decrease of the chances of ten-year survival in the presence of high scores, as scores above 6 represent zero chances of survival in the next 10 years.

The average of Charlson scores in relation to decades of age, analyzed step by step to identify significant differences between their averages (age categories and Charlson scores) indicates, depending on the established critical values, differences between pairs of compared averages, with significance *p* < 0.001 (Figure 3).

**Figure 4:**
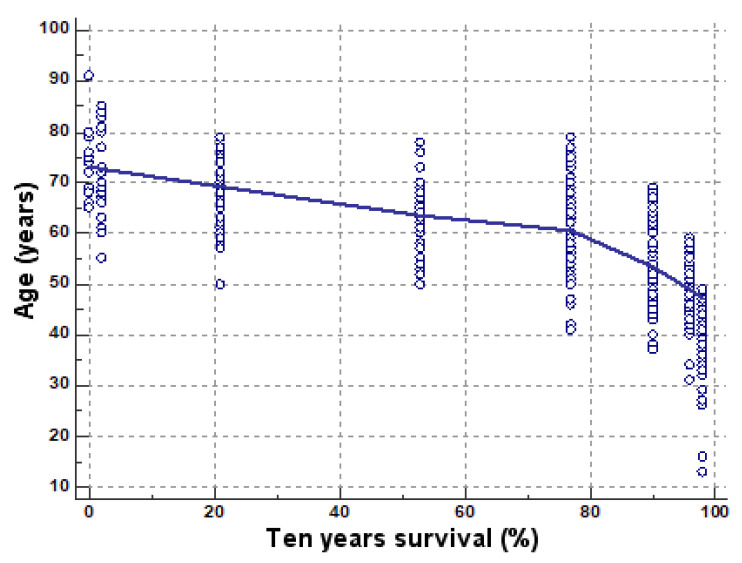
Negative correlation of the chance of survival by age (blue circles—cases that deviate from the average).

**Figure 5:**
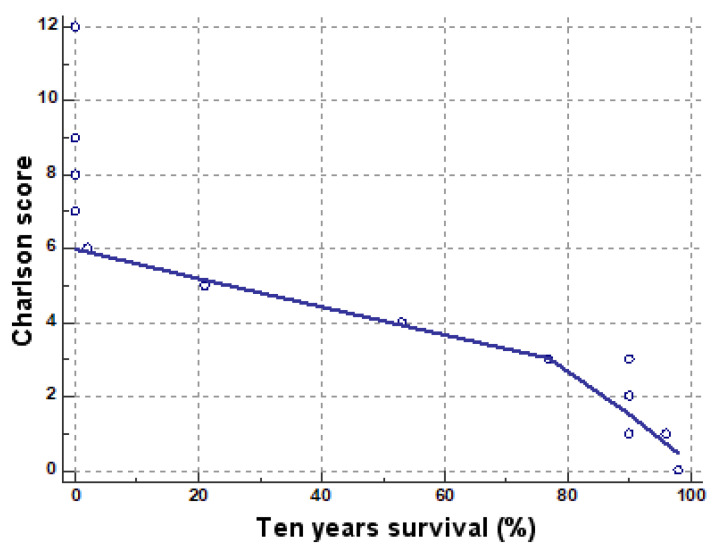
Negative correlation of the chance of survival with Charlson scores (blue circles—cases that deviate from the average).

The chances of survival at 10 years are moderately negatively correlated with age (sample size 594, r = −0.6706, *p* < 0.0001, 95% CI for r ranging between −0.7126 and −0.6238) (Figure 4). The chances of survival at 10 years strongly negatively correlate with the Charlson score (r = −0.9359, *p* < 0.0001, 95% CI for r ranging between −0.9452 and −0.9251) (Figure 5).

HT has the highest prevalence (62.28%, *n* = 370), with statistically significant variability depending on the groups of patients (Table 6). The relative risk (RR) for EC associated with HT is 1.4261 (95% CI: 1.2561 to 1.6191, *p* < 0.0001) compared to that of patients with benign (other) diagnoses. RR for EC and atypical EH is alike (*p* = 0.2676). RR for EC with HT is 1.6331 (95% CI: 1.3601 to 1.9609, *p* < 0.0001) compared to typical EH.

Out of a total of 594 patients, 113 patients (22.39%) presented DM, the highest percentage being found in cases with EC and typical EH (Table 7). The RR for the association of DM + EC compared to DM + other is 1.8342 (95% CI: 1.3053 to 2.5774, *p* = 0.0005). If assessing the risk created by the presence of DM and the occurrence of EC + atypical EH compared to DM + typical EH, an RR of 1.4848 is obtained, without statistical significance (95% CI: 0.5304 to 4.1565, *p* = 0.4516). If the risk created by the presence of DM and the occurrence of EC in relation to DM + typical EH is assessed, an RR of 1.4106 is obtained, without statistical significance (95% CI: 0.9544 to 2.0848, *p* = 0.0844).

Analysis of variance (ANOVA) for the presence of obesity in groups of patients enrolled after diagnosis (Table 8) and multiple comparisons (via the Tukey post-hoc test), indicates significant differences: between obesity in EC and atypical EH (95% CI: 0.00 to 0.63, *p* = 0.049); between obesity in EC and typical EH (95% CI: 0.30 to 0.57, *p* = 0.0001); between obesity in EC and that in other diagnoses (95% CI: 0.33 to 0.55, *p* = 0.049) (Table 9).

## 4. Discussion

In this bicentric retrospective cohort study of patients with endometrial lesions that has equitable clinical review, a knowledgeable effect on the overall survival has been proven through the measured medical comorbidities with the use of the validated age adjusted CCI. The zero-score CCI (representing the absence of comorbidities applied to the control group) indicates an average age of patients of 42 years; this average age increases to 51 years for patients who meet the value 1 of the Charlson score, to 58 years for those with a Charlson score of 2, and to 65 years for patients who have in addition to the underlying disease for which they are seeking hospital care during the analyzed period a CCI of 3. This discourages the clinician who faces patients over 65 with EC, because the life expectancy of these patients may be threatened, in addition to tumor disease, by the accumulation of associated diseases. Patients with more comorbidities may have a delayed diagnosis, resulting in a more advanced stage of the pathology [17]. Several previous studies have shown a lesser use of surgery and adjuvant therapies in patients with EC and multiple comorbidities [18,19,20].

The most common comorbidities in patients in the study were HT (62.28%), obesity (35.01%), and DM (22.89%), all being risk factors for tumor diseases in general. In fourth place in frequency are cardiovascular diseases, usually consecutive to the first three, and which worsen as the treatment of other chronic diseases is non-existent or inconsistent. These results are also present in the published literature [21].

At the same time, the association of DM with EC and atypical EH is constant, and the association of DM and typical EH is a proven risk, which further demonstrates that other endometrial lesions are influenced by high blood sugar [22].

In recent research, both the population-based and retrospective cohort studies have indicated that both pathologies, EC, and DM, were firmly linked with an HR of 1.81 (95% CI: 1.37–2.41) and had an increased correlation, with an age-adjusted HR of 1.85 (95% CI: 1.36–2.50) [23]. This study shows that the prevalence of DM in the study group exceeds the prevalence in the general population (12.4%) by a factor of almost two (22.39%). The risk presented by DM on the endometrium, depending on the cases included on diagnostic criteria, is significantly increased for EC, DM being present in high rates and comparable in all categories analyzed. Moreover, the results of this study emphasize the need for better detection of DM, because one out of nine women with DM might develop EC or atypical EH during their lifetime.

Obesity is also a threat to health in general and to reproductive health in particular; as with DM, there may be at least eight other obese women who may develop EC or atypical EH, which demonstrates the role of obesity in endometrial neoplasms. Obesity is firmly associated with the development of EC more than any other cancer type among women [24]. Results of a retrospective study of women with early EC show that women that are morbidly obese have a higher mortality rate in comparison to women with a normal body mass index (BMI), and 67% of these deaths were a result of noncancerous, obesity-related causes [25].

Health care providers such as oncologists and others are often reluctant to counsel patients with EC about obesity. A separate survey shows that of 108 women with EC only 29% reported being told by their health care provider about the link between obesity and the development of EC. The women who were counseled about obesity by their oncologists attempted to lose weight [26].

High blood pressure is a risk for EC compared to typical EH, and every woman with HT should increase her doctor’s alert, as there may be at least three other women with HT who may develop EC or atypical EH, which demonstrates the role of HT in endometrial neoplasms, as highlighted by other research [27]. HT is a strong risk factor for EC with a 61% increase in the RR as it is shown in meta-analysis of a published observational study, although the association was weaker in cohort studies (RR = 1.32) than among case-control studies (RR = 1.73) [27].

In our study, age was also validated as having a significant contribution to the overall survival factor, being incorporated into the Charlson comorbidity score to create a single accounting index for both age and medical comorbidity. The 10-year survival chances reach values on average of 95.8571% for women < 50 (SD 4.3712); these chances decreased to 82.3125% (SD 19.0037) for the decade of age 50–59 years, to 62.9061% (SD 28.3764) for the decade of age 60–69 years, to 35.9833% for the decade 70–79 years (SD 30.5811), and to 1.3846% (SD 0.9608) for patients > 80 years.

This study confirms that the presence of comorbidities correlates strongly positively with EC. Systemic manifestations, the appearance of symptoms and nonspecific signs that the patient presents, as well as the comorbidities associated with each case, play an important role in gynecologic practice. The patient’s prognosis depends not only on the EC but also on the context of all the associated pathologies that the patient has.

This study demonstrates the utility of CCI in establishing the clinical prognosis for patients with EC and EH in correlation with the individual comorbidities and with the status of healthcare services and medical research. It also shows that in order to intercept early stages of EC, a close monitoring of atypical EH is needed, with regular check-ups and ultrasound measurements, as this transition takes about 10 years in general, but in a group of cases, it might be very short. Our research proved the role of DM, HT and other cardiac diseases as negatively influencing comorbidities, a reason for which a multidisciplinary therapeutic approach is mandatory in order to increase the 5- and 10-year survival rates. The relationship between obesity and EC and EH has been proved once more. Therefore, the present study demonstrates the fact that the CCI is a very good prognostic, better than the values obtained by analyzing any of its variables alone, and that this score can be useful for gynecologic wards involved in the management of endometrial pathology.

As a shortcoming of this study, the authors have not performed a comparative evaluation of the patient’s characteristics of ACCI (age-adjusted CCI) ≤ median vs. ACCI > median because, due to the lack of information about the deceased patients, this analysis would have been redundant.

## 5. Conclusions

Data provided by this study clearly highlight that the cases with associated pathologies are statistically significant. The use of the CCI in endometrial lesions is necessary to compare the estimated risk of EC mortality with other medical conditions. Such comparison could help focus attention on the evolution of the disease and increase their overall survival and improve their quality of life, leading to better optimization of medical comorbidity, improved cancer management, and better estimation of the long-term potential of the effects of adjuvant treatments.

## Figures and Tables

**Table 1:** Analysis of the average age variance depending on the diagnosis (95% CI).

Dependent Variable: Age						
(I) Group	(J) Group	Difference (Average) (I-J)	SD	Sig.	Less or Equal to	Greater or Equal to
EC	Atypical EH	6.099	3.038	0.186	−1.73	13.93
	Typical EH	14.277 *	1.295	0.000	10.94	17.61
	Other	8.964 *	1.083	0.000	6.17	11.75
Atypical EH	EC	−6.099	3.038	0.186	−13.93	1.73
	Typical EH	8.178 *	3.059	0.039	0.30	16.06
	Other	2.865	2.976	0.771	−4.80	10.53
Typical EH	EC	−14.277 *	1.295	0.000	−17.61	−10.94
	Atypical EH	−8.178 *	3.059	0.039	−16.06	−0.30
	Other	−5.313 *	1.142	0.000	−8.25	−2.37
Other	EC	−8.964 *	1.083	0.000	−11.75	−6.17
	Atypical EH	−2.865	2.976	0.771	−10.53	4.80
	Typical EH	5.313 *	1.142	0.000	2.37	8.25

Legend: Sig—significance, Tukey’s HSD (honestly significant difference)—comparison of possible medium pairs, SD—standard deviation, EC—endometrial cancer, EH—endometrial hyperplasia, CI—confidence interval, * indicates statistical significance (*p* < 0.05).

**Table 2:** Distribution of comorbidities.

Diagnosis	Patients	
	Number	%
Diabetes	133	22.39
Obesity	208	35.01
High blood pressure	370	62.28
Dyslipidemia	46	7.74
Hepatitis B or C virus	15	2.52
Veins disorders	59	9.93
Cardiovascular	110	18.51
Renal insufficiency	4	0.67
Asthma, bronchopneumonia	15	2.52
Locomotor, sensory, and cognitive deficits	30	5.05

**Table 3:** Comorbidities.

Comorbidity	Other		EC		Atypical EH		Typical EH		Total	
	No	%	No	%	No	%	No	%	No	%
Uncomplicated diabetes	41	39.42	35	33.65	2	1.92	26	25.00	104	17.51
Localized solid tumor	10	25.00	10	25.00	0	0.00	20	50.00	40	6.73
Diabetes mellitus with organ failure	10	34.48	14	48.28	1	3.45	4	13.79	29	4.88
Congestive heart failure	11	35.48	17	54.84	1	3.23	2	6.45	31	5.22
Mild liver damage	14	28.00	9	18.00	14	28.00	13	26.00	50	8.42
Chronic moderate or severe kidney disease	3	30.00	6	60.00	0	0.00	1	10.00	10	1.68
Moderate to severe liver damage	4	57.14	2	28.57	0	0.00	1	14.29	7	1.18
Stroke or transient ischemic attack	3	7.50	19	47.50	12	30.00	6	15.00	40	6.73
Hemiplegia	0	0.00	3	100.00	0	0.00	0	0.00	3	0.51
Peripheral arterial disease	1	25.00	2	50.00	1	25.00	0	0.00	4	0.67
Metastatic solid tumor	2	100.00	0	0.00	0	0.00	0	0.00	2	0.34
Ulcerative peptic disease	1	25.00	2	50.00	0	0.00	1	25.00	4	0.67
Dementia	1	11.11	4	44.44	3	33.33	1	11.11	9	1.52

Legend: EC—endometrial cancer, EH—endometrial hyperplasia.

**Table 4:** Charlson scores and chances of survival at 10 years (*p* < 0.0001).

Ten-Year Survival %									
Charlson Score	0	2	21	53	77	90	96	98	Total (no/%)
0	00.0% RT	00.0% RT	00.0% RT	00.0% RT	00.0% RT	00.0% RT	00.0% RT	130100.0% RT	130/21.9
	0.0% CT	0.0% CT	0.0% CT	0.0% CT	0.0% CT	0.0% CT	0.0% CT	100.0% CT	
	0.0% GT	0.0% GT	0.0% GT	0.0% GT	0.0% GT	0.0% GT	0.0% GT	21.9% GT	
1	00.0% RT	00.0% RT	00.0% RT	00.0% RT	00.0% RT	11.0% RT	9699.0% RT	00.0% RT	97/16.3
	0.0% CT	0.0% CT	0.0% CT	0.0% CT	0.0% CT	0.8% CT	100.0% CT	0.0% CT	
	0.0% GT	0.0% GT	0.0% GT	0.0% GT	0.0% GT	0.2% GT	16.2% GT	0.0% GT	
2	00.0% RT	00.0% RT	00.0% RT	00.0% RT	00.0% RT	116100.0% RT	00.0% RT	00.0% RT	116/19.5
	0.0% CT	0.0% CT	0.0% CT	0.0% CT	0.0% CT	97.5% CT	0.0% CT	0.0% CT	
	0.0% GT	0.0% GT	0.0% GT	0.0% GT	0.0% GT	19.5% GT	0.0% GT	0.0% GT	
3	00.0% RT	00.0% RT	00.0% RT	00.0% RT	8297.6% RT	22.4% RT	00.0% RT	00.0% RT	84/14.1
	0.0% CT	0.0% CT	0.0% CT	0.0% CT	100.0% CT	1.7% CT	0.0% CT	0.0% CT	
	0.0% GT	0.0% GT	0.0% GT	0.0% GT	13.8% GT	0.3% GT	0.0% GT	0.0% GT	
4	00.0% RT	00.0% RT	00.0% RT	79100.0% RT	00.0% RT	00.0% RT	00.0% RT	00.0% RT	79/13.3
	0.0% CT	0.0% CT	0.0% CT	100.0% CT	0.0% CT	0.0% CT	0.0% CT	0.0% CT	
	0.0% GT	0.0% GT	0.0% GT	13.3% GT	0.0% GT	0.0% GT	0.0% GT	0.0% GT	
5	00.0% RT	00.0% RT	47100.0% RT	00.0% RT	00.0% RT	00.0% RT	00.0% RT	00.0% RT	47/7.9
	0.0% CT	0.0% CT	100.0% CT	0.0% CT	0.0% CT	0.0% CT	0.0% CT	0.0% CT	
	0.0% GT	0.0% GT	7.9% GT	0.0% GT	0.0% GT	0.0% GT	0.0% GT	0.0% GT	
6	00.0% RT	25100.0% RT	00.0% RT	00.0% RT	00.0% RT	00.0% RT	00.0% RT	00.0% RT	25/4.2
	0.0% CT	100.0% CT	0.0% CT	0.0% CT	0.0% CT	0.0% CT	0.0% CT	0.0% CT	
	0.0% GT	4.2% GT	0.0% GT	0.0% GT	0.0% GT	0.0% GT	0.0% GT	0.0% GT	
7	4100.0% RT	00.0% RT	00.0% RT	00.0% RT	00.0% RT	00.0% RT	00.0% RT	00.0% RT	4/0.7
	25.0% CT	0.0% CT	0.0% CT	0.0% CT	0.0% CT	0.0% CT	0.0% CT	0.0% CT	
	0.7% GT	0.0% GT	0.0% GT	0.0% GT	0.0% GT	0.0% GT	0.0% GT	0.0% GT	
8	9100.0% RT	00.0% RT	00.0% RT	00.0% RT	00.0% RT	00.0% RT	00.0% RT	00.0% RT	9/1.5
	56.2% CT	0.0% CT	0.0% CT	0.0% CT	0.0% CT	0.0% CT	0.0% CT	0.0% CT	
	1.5% GT	0.0% GT	0.0% GT	0.0% GT	0.0% GT	0.0% GT	0.0% GT	0.0% GT	
9	2100.0% RT	00.0% RT	00.0% RT	00.0% RT	00.0% RT	00.0% RT	00.0% RT	00.0% RT	2/0.3
	12.5% CT	0.0% CT	0.0% CT	0.0% CT	0.0% CT	0.0% CT	0.0% CT	0.0% CT	
	0.3% GT	0.0% GT	0.0% GT	0.0% GT	0.0% GT	0.0% GT	0.0% GT	0.0% GT	
12	1100.0% RT	00.0% RT	00.0% RT	00.0% RT	00.0% RT	00.0% RT	00.0% RT	00.0% RT	1/0.2
	6.2% CT	0.0% CT	0.0% CT	0.0% CT	0.0% CT	0.0% CT	0.0% CT	0.0% CT	
	0.2% GT	0.0% GT	0.0% GT	0.0% GT	0.0% GT	0.0% GT	0.0% GT	0.0% GT	
Total no/%	16/ 2.7	25/ 4.2	47/ 7.9	79/ 13.3	82/ 13.8	119/ 20	96/ 16.2	130/ 21.9	594/ 100

**Table 5:** ANOVA one way variance analysis (*p* < 0.001) for chances of survival and Charlson score (*p* < 0.05).

Factor	*N*	Average	Standard Deviation	Different from Factor Number
(1) 0	130	98.0000	0.0000	(2)(3)(4)(5)(6)(7)(8)(9)(10)(11)
(2) 1	97	95.9381	0.6092	(1)(3)(4)(5)(6)(7)(8)(9)(10)(11)
(3) 2	116	90.0000	0.0000	(1)(2)(4)(5)(6)(7)(8)(9)(10)(11)
(4) 3	84	77.3095	1.9938	(1)(2)(3)(5)(6)(7)(8)(9)(10)(11)
(5) 4	79	53.0000	0.0000	(1)(2)(3)(4)(6)(7)(8)(9)(10)(11)
(6) 5	47	21.0000	0.0000	(1)(2)(3)(4)(5)(7)(8)(9)(10)(11)
(7) 6	25	2.0000	0.0000	(1)(2)(3)(4)(5)(6)(8)(9)(10)
(8) 7	4	0.0000	0.0000	(1)(2)(3)(4)(5)(6)(7)
(9) 8	9	0.0000	0.0000	(1)(2)(3)(4)(5)(6)(7)
(10) 9	2	0.0000	0.0000	(1)(2)(3)(4)(5)(6)(7)
(11) 12	1	0.0000	0.0000	(1)(2)(3)(4)(5)(6)

**Table 6:** Frequency of hypertension in the study groups.

Group	Arterial Hypertension	Percent %
Other	166	56.46
EC	124	81.04
Atypical EH	9	64.28
Typical EH	71	53.38
Total number/%	370/100.0	62.28

Legend: EC—endometrial cancer, EH—endometrial hyperplasia.

**Table 7:** Percentage of the association of diabetes with gynecologic diagnosis.

Association of DM + Diagnosis	Yes	No	% DM	Total
DM + EC	49	104	32.02	153
DM + Atypical EH	3	11	21.42	14
DM + Typical EH	30	103	22.45	133
DM + other	51	243	17.34	294
Total	133	461	22.39	594

Legend: DM—diabetes mellitus, EC—endometrial cancer, EH—endometrial hyperplasia.

**Table 8:** Number of cases and percentage of obesity in the study groups (*p* < 0.0001).

Group	No. of Patients	%
Other	69	23.46
	100.0% RT	
	33.2% CT	
	33.2% GT	
EC	103	67.32
	100.0% RT	
	49.5% CT	
	49.5% GT	
Atypical EH	5	35.71
	100.0% RT	
	2.4% CT	
	2.4% GT	
Typical EH	31	23.3
	100.0% RT	
	14.9% CT	
	14.9% GT	
Total	208	100

Legend: EC—endometrial cancer, EH—endometrial hyperplasia.

**Table 9:** Variance of obesity in the study groups.

Tukey HSD Dependent Variable Obesity						
(I) Group	(J) Group	Av. Diff. (I-J)	SE	Sig.	95% Confidence Interval	
					≤	≥
EC	Atypical EH	0.316 *	0.122	0.049	0	0.63
	Typical EH	0.437 *	0.052	0	0.3	0.57
	other	0.440 *	0.044	0	0.33	0.55
Atypical EH	EC	−0.316 *	0.122	0.049	−0.63	0
	Typical EH	0.121	0.123	0.762	−0.2	0.44
	Other	0.124	0.12	0.729	−0.18	0.43
Typical EH	EC	−0.437 *	0.052	0	−0.57	−0.3
	Atypical EH	−0.121	0.123	0.762	−0.44	0.2
	Other	0.004	0.046	1	−0.12	0.12
Other	EC	−0.440 *	0.044	0	−0.55	−0.33
	Atypical EH	−0.124	0.12	0.729	−0.43	0.18
	Typical EH	−0.004	0.046	1	−0.12	0.12

* The average difference is significant at 0.05.EC—endometrial cancer, EH—endometrial hyperplasia, SE—standard error, Av. Diff—average difference.

## Data Availability

All data are available in the archives (database) of the Emergency Clinical County Hospitals of Arad and Timis, Romania.
